# Exploration of different yogic states for a better understanding of consciousness: An electromagnetic perspective

**DOI:** 10.4103/0973-6131.43545

**Published:** 2008

**Authors:** Ravi Prakash, Shashi Prakash, Om Prakash, Nitin Bhatt

**Affiliations:** Central Institute of Psychiatry, Ranchi, India; 1 Tata Consultancy Services, Pune, India; 2 Biotechnology Department, Delhi College of Engineering, Delhi, India

**RESPECTED SIR,**

Many diseases are complicated by impaired consciousness, which is a difficult variable for clinical neurologists to control. Thus, in addition to physicists, mathematicians, and cognitive neuroscientists, clinical neurologists also seek to have a clearer concept of consciousness. Although the use of sophisticated and new technology to explore consciousness is relatively new, a significant amount of literature on various theories of consciousness already exists. Literature studies involve either electroencephalogram (EEG), functional Magnetic Resonance Imaging (fMRI) and Positron Emission Tomography (PET) scans, in which the neural correlates of various states of consciousness are recorded, or the construction of various mathematical models based on available data.[1]

However, it is the authors' opinion that studies conducted so far have been limited to the exploration of states of wakefulness, sleep, and pathologically altered consciousness. Hence, it is desirable to study purer states of consciousness by probing various yogic states, a topic on which little scientific literature exists. Some states like yoga nidra have been adequately studied, and the results have been convincing in terms of electrophysiological and neurochemical changes in this trance state.[2] The yogic and religious literatures are full of description of states which are unique in addition to being novel. For example, Vihangam Yogic meditation procedure, a commonly practiced meditation in northern India, mentions 6 states of consciousness, which are the *Sthul awastha, Sookshma awastha, Karana awastha, Mahakarana awastha, Kaivalya awastha* and the *Hamsa awastha*. A few of them deserve a mention here. Turiya awastha has been for example mentioned as a state of higher awareness where the meditatior becomes aware of the dimensions of nature and self which are not possible in awake state. Kaivalya and Samadhi states have been described as states where the person comes in communion with the god, nature, supreme consciousness and so on[3,6]. *Hamsa awastha* is a unique state mentioned over all the yogic texts, with unique features. His holiness Sadguru Sadafal dev ji Maharaj, founder of Vihangam Yogic organization, gave a beautiful description of consciousness in this state in the form of a bird, which flies on his own, a term known a *Vihangama* or flight.[6] These states may seem abstract and difficult to study. But there are several experiences associated with these states, which can be more easily approached. For example, perception of inner light is one such experience, whose EEG findings have been studied and yielded important objective findings like blocking of alpha rhythm[7]. The recognition of the need to explore purer states of consciousness such as various meditational states is not new, and has already been pointed out by Susan Pockett, who has been working to find electromagnetic correlates of consciousness in the brain, and has postulated a theory of consciousness.[4]

There could be many scientific approaches to studying these states. However, we want to stress electromagnetic approaches because of the following reasons:

1) The electromagnetic events of brain are closely linked to the underlying cognitive and perceptual processes. This is the reason that much of the cognitive and perceptual sciences researches are based on investigating the electoromagnetic events of brain.
2) We have acquired several investigative tools for measurement of electromagnetic aspects of brain, including EEG, Event related potentials (ERP), exploratory repetitive transcranial magnetic stimulation (rTMS) etc.

Susan has pointed out various spatial patterns of EEG found in these states, as possible electromagnetic correlates to the states of consciousness. As per her view consciousness increases the complexity of electromagnetic field of brain, thus the spatial complexity increases in Yogic states. Our group has been working on the effects of the electromagnetic field of the brain on the electrical states of individual neurons.[5] Analyses of human EEG and animal intraneuronal data have revealed that the electromagnetic field of the brain can have very obvious impacts on the firing of individual neurons. However, we need to fill in lacunae in current literature by collecting data on electromagnetic field configurations from states of more concentrated consciousness, such as those of the higher yogic states.

Yoga has proved to be invaluable in helping patients suffering from various diseases and is an equally interesting subject of study for clinical trials as it is for basic research of consciousness. The authors believe that the pursuit of the definition of consciousness will be greatly aided by the study of higher states of awareness including *Turiya awastha, Samadhi awastha,* and *Hansa awastha*. Thus, using this much awaited platform of the International Journal of Yoga, we appeal to all scientists and mathematicians to orient themselves to pursue the study of higher levels of awareness.
